# Multienzyme Immobilization on PVDF Membrane via One-Step Mussel-Inspired Method: Enhancing Fouling Resistance and Self-Cleaning Efficiency

**DOI:** 10.3390/membranes14100208

**Published:** 2024-09-27

**Authors:** Jéssica Mulinari, Diane Rigo, Carolina Elisa Demaman Oro, Alessandra Cristina de Meneses, Guilherme Zin, Rafael Vidal Eleutério, Marcus Vinícius Tres, Rogério Marcos Dallago

**Affiliations:** 1 TransferTech Gestão de Inovação, Erechim 99700-420, Brazil; dianerigo@hotmail.com (D.R.); or 044079@aluno.uricer.edu.br (C.E.D.O.); acmeneses@transfertech.com.br (A.C.d.M.); gzin@transfertech.com.br (G.Z.); 2 Department of Food and Chemical Engineering, Universidade Regional Integrada do Alto Uruguai e das Missões (URI), 1621 Sete de Setembro Av., Centro, Erechim 99709-910, Brazil; dallago@uricer.edu.br; 3 Graduate Program in Materials Science and Engineering (PGMAT), Federal University of Santa Catarina (UFSC), Florianópolis 88040-900, Brazil; rafaelvebr@gmail.com; 4 Laboratory of Agroindustrial Processes Engineering (LAPE), Federal University of Santa Maria (UFSM), Cachoeira do Sul 96503-205, Brazil

**Keywords:** lipase immobilization, amylase immobilization, protease immobilization, membrane fouling, membrane separation process, polydopamine, dairy wastewater

## Abstract

Immobilizing different enzymes on membranes can result in biocatalytic active membranes with a self-cleaning capacity toward a complex mixture of foulants. The membrane modification can reduce fouling and enhance filtration performance. Protease, lipase, and amylase were immobilized on poly(vinylidene fluoride) (PVDF) microfiltration membranes using a polydopamine coating in a one-step method. The concentrations of polydopamine precursor and enzymes were optimized during the immobilization. The higher hydrolytic activities were obtained using 0.2 mg/mL of dopamine hydrochloride and 4 mg/mL of enzymes: 0.90 mg_starch_/min·cm^2^ for amylase, 10.16 nmol_tyrosine_/min·cm^2^ for protease, and 20.48 µmol_p-nitrophenol_/min·cm^2^ for lipase. Filtration tests using a protein, lipid, and carbohydrate mixture showed that the modified membrane retained 41%, 29%, and 28% of its initial water permeance (1808 ± 39 L/m^2^·h·bar) after three consecutive filtration cycles, respectively. In contrast, the pristine membrane (initial water permeance of 2016 ± 40 L/m^2^·h·bar) retained only 23%, 12%, and 8%. Filtrations of milk powder solution were also performed to simulate dairy industry wastewater: the modified membrane maintained 28%, 26%, and 26% of its initial water permeance after three consecutive filtration cycles, respectively, and the pristine membrane retained 34%, 21%, and 7%. The modified membrane showed increased fouling resistance against a mixture of foulants and presented a similar water permeance after three cycles of simulated dairy wastewater filtration. Membrane fouling is reduced by the immobilized enzymes through two mechanisms: increased membrane hydrophilicity (evidenced by the reduced water contact angle after modification) and the enzymatic hydrolysis of foulants as they accumulate on the membrane surface.

## 1. Introduction

Despite the extensive study of membrane systems, their application in wastewater treatment faces limitations, primarily due to fouling issues. Fouling is a problem for all types of membranes, both organic and inorganic. However, in polymeric membranes, it is more pronounced due to the hydrophobic nature of polymers. Polyvinylidene fluoride (PVDF) membranes are the most widely used in various filtration applications due to their high chemical resistance, thermal stability, and mechanical strength [1]. These characteristics make PVDF membranes ideal for microfiltration and ultrafiltration in water and wastewater treatment. However, in order to have a broader application of these membranes, several aspects must be improved, mainly their durability and cost-effectiveness in industrial applications. These two aspects are intimately related to fouling, since it leads to a decline in permeate flux, which reduces system efficiency, raises the energy consumption, and increases the frequency of membrane cleaning operations, which are all factors that can shorten the membrane’s lifespan and raise the overall process costs [2].

Several methods to enhance the antifouling properties of membranes have been developed, mainly focusing on modifying the hydrophilicity, surface charge, or affinities of the membrane surface [3]. One promising strategy involves immobilizing enzymes on the membrane surface, creating an “active” membrane that can degrade fouling during filtration [4,5,6,7]. The immobilized enzymes give the membrane a self-cleaning capability, minimizing the need for chemical use during the cleaning process. Conventional cleaning of a fouled membrane often requires harsh or environmentally harmful chemicals such as strong oxidants like hypochlorite, alkali, or acid solutions [3,8]. The concept of self-cleaning membranes relies on immobilizing enzymes on the membrane surface, bringing the enzymes into direct contact with foulants and enabling the enzymatic removal of the fouling layer through hydrolysis [3].

Most enzyme immobilization methods involve several chemicals (such as EDC/NHS chemistry) or toxic compounds (such as glutaraldehyde), aside from being multistep and time-consuming. Greener and easier immobilization methods are crucial to boost this technology. A polydopamine (PDA) coating can be a competitive alternative to the usual methods for multiple enzyme immobilization. The development of PDA coatings was inspired by the adhesive capacity of marine mussels, which adhere to various substrates by secreting L-3,4-dihydroxyphenylalanine (L-DOPA) and its derivative dopamine [9,10,11]. Through dopamine polymerization, thin PDA layers can successfully modify a broad spectrum of inorganic and organic materials [12,13,14,15]. PDA coatings can enhance the antifouling properties of membranes by increasing their hydrophilicity [13,16,17]. Additionally, the PDA layer contains various reactive groups such as imine, amine, and catechol, which enable it to bond different materials and molecules (including enzymes) to the membrane [18,19,20,21,22]. Dopamine polymerization and enzyme (or multienzyme) immobilization can be performed simultaneously in only one step, which results in a lower consumption of chemicals and a consequent lower wastewater generation, besides a reduced immobilization time [23].

The immobilization of multiple enzymes on the membranes is essential to improve the filtration of complex solutions such as wastewater. Dairy wastewater, for example, contains high concentrations of carbohydrates, proteins, and fats [24,25]. Therefore, the immobilization of amylase for the hydrolysis of starch, protease for the hydrolysis of protein, and lipase for the hydrolysis of oils and fats can be a solution to decrease membrane fouling and improve the filtration performance during the filtration of dairy wastewater. Based on this, this study evaluated a one-step method using a PDA coating to immobilize an α-amylase (Termamyl Brew Q), a protease (Alcalase 2.4 L), and a lipase (Eversa Transform 2.0) on PVDF microfiltration membranes. The immobilization conditions, such as dopamine and enzyme concentrations, were evaluated to develop a membrane with high hydrolytic activities. The enzymatically active membrane was applied to the filtration of a mixture of substrates (starch, protein, and oil) and the filtration of a milk powder solution simulating dairy wastewater to evaluate its fouling-degrading and self-cleaning capacity.

## 2. Materials and Methods

### 2.1. Chemicals and Materials

The selected enzymes were amylase Termamyl Brew Q (produced by *Bacillus licheniformis*), lipase Eversa Transform 2.0 (ET2) (produced by *Aspergillus oryzae*), and protease Alcalase 2.4 L FG (produced by *Bacillus licheniformis*). The enzyme’s liquid solutions were kindly donated by Novozymes. The membrane used was a PVDF microfiltration flat-sheet membrane (NADIR MV020 T) with average pores of 200 nm.

The following chemicals were used: dopamine hydrochloride (DA, C_8_H_11_NO_2_·HCl, Sigma-Aldrich, Saint Louis, MO, USA), sodium dodecyl sulfate 90% (SDS, C_12_H_25_SO_4_Na, Synth, Diadema, Brazil), casein 90% (Synth, Brazil), trichloroacetic acid (TCA, C_2_HCl_3_O_2_, Neon, Suzano, Brazil), tyrosine (C_9_H_11_NO_3_, Synth, Brazil), soluble starch 99.6% (Synth, Brazil), potassium iodate (KI, Synth, Brazil), iodine (I_2_, Synth, Brazil), hydrochloric acid 37% (HCl, Neon, Brazil), p-nitrophenyl acetate (C_8_H_7_NO_4_, p-NPA, Sigma-Aldrich, USA), acetonitrile (C_2_H_3_N, Neon, Brazil), and p-nitrophenol (C_6_H_5_NO_3_, Neon, Brazil). The Tris-HCl buffer was prepared using tris(hydroxymethyl)aminomethane (C_4_H_11_NO_3_, Neon, Brazil) and HCl 37% (HCl, Neon, Brazil). For the sodium phosphate buffer, sodium phosphate monobasic (NaH_2_PO_4_, Neon, Brazil) and sodium phosphate dibasic (Na_2_HPO_4_, Neon, Brazil) were used. The sodium-citrate buffer was prepared using citric acid (C_6_H_8_O_7_, Synth, Brazil) and disodium phosphate heptahydrate (Na_2_HPO_4_·7H_2_O). For the acetate buffer, sodium acetate (C_2_H_3_NaO_2_, Synth, Brazil), acetic acid (C_2_H_4_O_2_, Neon, Brazil), and HCl 37% (HCl, Neon, Brazil) were used. All chemicals were of analytical grade and were used as received without additional purification. Refined soybean oil (Soya, Sorriso, Brazil) and milk powder (Aurora, Brazil) were purchased from a local market.

### 2.2. Enzyme Preparation

Dialysis was conducted for 120 h using a 0.05 M sodium phosphate buffer at pH 6.0 to remove impurities and stabilizers from the commercial solutions. After dialysis, the solutions of purified enzymes were frozen at −20 °C and lyophilized for 48 h (Edwards Modulyo 4K, Manchester, UK). The resulting powders were stored at 4 °C until use. The protein content of the commercial solutions and lyophilized enzymes was determined by the Kjeldahl method, considering a factor of 6.25.

The hydrolytic activity of the commercial solution and the lyophilized enzymes were determined to compare the enzymes’ activities before and after purification and lyophilization. The amylase activity was determined by measuring the reduction in the binding capacity between starch and iodine, according to the method described by Fuwa [26]. For this, 0.3 mL of the diluted enzyme was added to 0.7 mL of a 0.3% (*w*/*v*) soluble starch in 0.25 M acetate buffer at pH 5. After incubation at 30 °C for 10 min, the reaction was stopped by adding 4 mL of 0.2 M HCl. Then, 0.5 mL of iodine reagent was added. A control sample was prepared by replacing the enzyme solution with distilled water. Quantification was conducted using a spectrophotometer (Pró-Análise UV-1600, Porto Alegre, Brazil) at a wavelength of 550 nm, with a calibration curve using different concentrations of starch and distilled water as the blank. The hydrolytic activity of the amylase was calculated in terms of the mass of starch hydrolyzed per minute of reaction per unit mass of enzyme added (mg_starch_/min·mg_enzyme_).

The lipase activity was determined according to the method used by De Yan et al. [27], in which the hydrolysis of p-NPA to p-nitrophenol is monitored spectrophotometrically. For this, 0.1 mL of 0.01 M p-NPA in acetonitrile was added to 5.6 mL of 0.05 M Tris-HCl buffer at pH 7.5 and 0.1 mL of diluted enzyme. The mixture was kept at 40 °C for 3 min. The reaction was carefully timed from the addition of the p-NPA solution to the spectrometric readings at 405 nm. Absorbance values were converted to µmol/L of p-nitrophenol by comparing them to a previously constructed calibration curve. Enzymatic activity was expressed in µmol of product formed per minute per unit mass of enzyme (µmol_p-nitrophenol_/min·mg_enzyme_).

The hydrolytic activity of the protease was determined through the enzymatic hydrolysis of casein, according to the methods proposed by Kunitz [28] and Walter [29] with modifications. When hydrolyzed, casein releases tyrosine, which can be determined spectrophotometrically. First, a 1% (*w*/*v*) casein solution in 0.05 M citrate-phosphate buffer at pH 7.0 was prepared and incubated at 40 °C for 10 min. A volume of 10 mL of this substrate was used for the assay, along with 100 µL of diluted enzyme, which were incubated at 50 °C and 150 rpm. After 30 min, 2 mL aliquots were removed from the reaction mixture and added to centrifuge tubes containing 4 mL of 10% (*v*/*v*) TCA. The tubes were kept at 8 °C for 1 h and then centrifuged for 6 min. The clear supernatant was used for spectrophotometric readings at a wavelength of 275 nm. Enzymatic activity was determined in triplicate by quantifying the acid-soluble peptides. Absorbance values were converted to mmol/L of tyrosine by comparing them to a previously constructed calibration curve. Enzymatic activity was expressed in mmol of product formed per minute per unit mass of enzyme (mmol_tyrosine_/min·mg_enzyme_).

### 2.3. Multi-Enzyme Immobilization

Membranes of 1 cm × 1 cm were used for the enzyme immobilization. Before the tests, the membranes were washed with ethanol for 1 min and twice with deionized water for 5 min, and then were stored in deionized water until use. A one-step immobilization method was used in which the membrane was functionalized with the ligand (polydopamine), and the enzymes were immobilized simultaneously, as proposed by Mulinari et al. [23]. A central composite rotatable design (CCRD) 2^2^ was performed by varying the DA concentration (0.2–0.8 mg/mL) and the enzyme concentrations (2.6–5.4 mg/mL) in the solution to determine the best immobilization conditions based on the amylase, lipase, and protease hydrolytic activity of the membrane. The enzyme concentration (EN) was the total concentration of the three enzymes added in equal amounts. For example, an EN of 3 mg/mL means adding 1 mg/mL of each enzyme. Both dopamine and the enzymes were dissolved in 0.05 M Tris-HCl buffer at pH 8.5. The membrane was immersed in 10 mL of the immobilization solution for 12 h at 150 rpm before being washed with deionized water to remove the excess enzyme and dopamine.

The hydrolytic activity of the immobilized enzymes was determined as described in Section 2.2 by immersing the membranes in 10 mL of the substrate solution. The activity of the immobilized enzymes was expressed per membrane area. The software Statistica 8.0 was used for the statistical analyses of the CCRD. The best immobilization condition was used to modify a 19 cm × 9 cm membrane with a useful area of 162 cm^2^ for use in the microfiltration tests.

### 2.4. Microfiltration Tests

The filtration performance of the membrane modified by the best immobilization conditions was compared to the pristine membrane and the membrane modified with polydopamine only. The fouling resistance and self-cleaning capability of the membranes were assessed by the filtration of a mixed solution of starch (0.75 g/L), casein (0.5 g/L), and soybean oil (1 g/L) following previously reported methodologies [3,7]. A milk powder solution (2 g/L) was also filtered to simulate dairy industry wastewater. For the mixed solution, starch and casein were dissolved in 400 mL of distilled water by boiling it for 10 min, and soybean oil was emulsified within 400 mL of a 2 mM SDS aqueous solution by stirring magnetically at 1500 rpm for 10 min. The solutions were mixed, and the volume was adjusted to 1 L.

In the filtration experiment, deionized water was first filtered at 0.5 bar with a retentate flow rate of 0.5 L/min to determine the initial pure water flux of the membrane (J_0_). Following this, three filtration steps, each lasting 2 h, were conducted using the mixed or milk powder solution in a cross-flow configuration, with complete recirculation of the retentate and permeate back to the feed reservoir. After each filtration step, a 10-min cleaning was performed at 0.5 bar and a retentate flow rate of 0.5 L/min using distilled water. The pure water flux (J) was measured again to assess fouling in each cycle, expressed as the percentage of flux remaining after each filtration.

### 2.5. Characterization

The hydrophilicity of the pristine and modified membranes was evaluated through the static pure water contact angle (WCA) determined by the sessile drop method using a goniometer (KRÜSS DAS 25, Hamburg, Germany). The surface chemistry of the membranes was evaluated through infrared spectroscopy with the attenuated total reflection method (ATR-FTIR) in a spectrophotometer (Agilent Cary 630, Santa Clara, CA, USA) from 4000 to 650 cm^−1^. The lyophilized enzymes were also analyzed through ATR-FTIR.

## 3. Results

### 3.1. Enzymes Preparation

The enzymes were dialyzed and lyophilized to remove stabilizers and impurities from the crude extract and to concentrate the enzyme of interest. Table 1 shows the protein content and the specific hydrolytic activity of each enzyme before and after purification/concentration.

Based on the protein content, it is evident that the process efficiently concentrated the enzymes, which showed purities of approximately 66%, 54%, and 60% for amylase, lipase, and protease, respectively. Moreover, the purification/concentration method used did not negatively affect the hydrolytic activity of the enzymes.

### 3.2. Multi-Enzyme Immobilization

The membranes were functionalized using a one-step method with polydopamine and the enzymes. Table 2 presents the results for the hydrolytic activities of the immobilized amylase, lipase, and protease according to the CCRD 2^2^.

**Figure 1:**
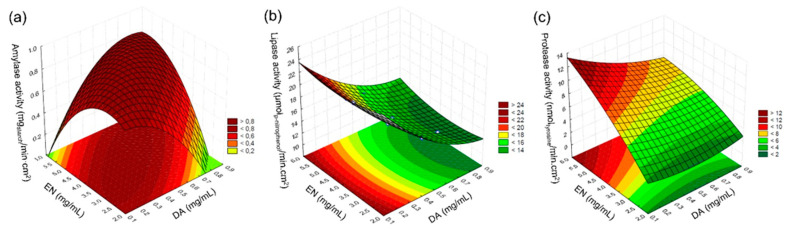
Response surfaces representing the behavior of the hydrolytic activity of the immobilized (**a**) amylase, (**b**) lipase, and (**c**) protease.

Amylase activity peaked at the central points of the design (assays 9 to 11, using 0.5 mg/mL of DA and 1.33 mg/mL of each enzyme, totaling 4 mg/mL of EN). The Supplementary Materials present the analysis of variance (ANOVA) for the results obtained in the experimental design, considering a *p*-value < 0.05 (Table S1). The response surface of Figure 1a illustrates the behavior of the immobilized amylase within the tested concentration ranges. An R^2^ of 0.89 was obtained for the model. It can be noted that the activity of the immobilized amylase was directly influenced by the concentration of DA and EN: for higher concentrations of DA, a higher concentration of EN was required to achieve the maximum hydrolytic activity, while for lower concentrations of DA, lower concentrations of EN were sufficient to reach the maximum activity. This occurred because polydopamine at high concentrations relative to the enzyme can cover the entire enzymatic structure, hindering the substrate’s access to the active site.

Lipase activity increased when 0.2 mg/mL of DA and 4.0 mg/mL of EN were used (assay 5). Table S2 presents the ANOVA for the lipase activity according to the CCRD 2^2^. The results are represented in the surface response of Figure 1b. An R^2^ of 0.99 was obtained for the model. As observed for amylase, lower DA concentrations led to higher lipase hydrolytic activity, possibility due to the covering of the enzymes by PDA if a higher DA concentration is used; however, the enzyme concentration did not affect the lipase activity. This fact suggests that lipase is still active, even in lower enzyme concentrations.

The highest protease activity was achieved using 0.3 mg/mL of DA and 1.67 mg/mL of each enzyme, totaling 5 mg/mL (assay 3). Table S3 shows the ANOVA for the protease hydrolytic activity obtained in the experimental design. The surface response of Figure 1c represents the behavior of the immobilized protease within the concentrations tested. An R^2^ of 0.95 was obtained for the model. Lower DA and higher EN concentrations for the protease led to higher hydrolytic activities. Mulinari et al. [7] had similar results for lipase immobilization on ceramic membranes using the same immobilization method.

Since the three enzymes had different behaviors and presented higher hydrolytic activities in different immobilization conditions, additional tests were performed using each enzyme’s optimal DA and EN concentrations (Table 3). Tukey’s test showed that immobilized amylase activity using 0.2 or 0.5 mg/mL of DA did not have a significant difference (*p* < 0.05). Therefore, immobilized amylase and lipase activities were higher using 0.2 mg/mL of DA and 4.0 mg/mL of EN, which was the immobilization condition selected to modify the membrane used in the characterization and filtration tests.

### 3.3. Membrane Characterization

**Figure 2:**
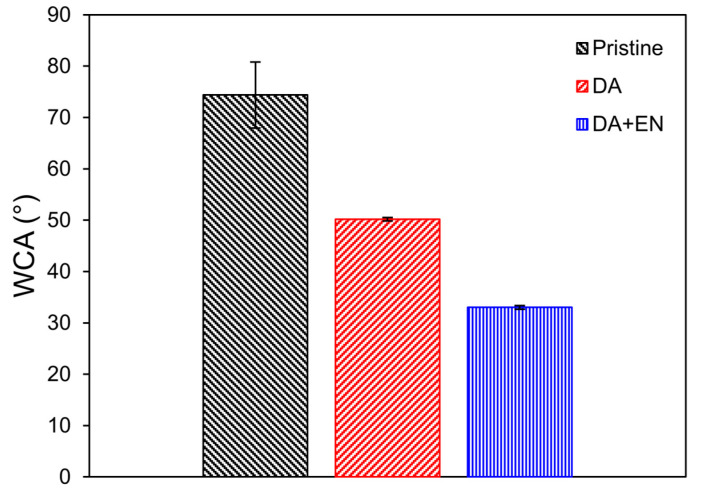
WCA of the pristine, PDA-coated (0.2 mg/mL), and PDA + EN (0.2 mg/mL of DA and 4 mg/mL of EN) modified membranes.

The WCA of the membranes was measured to determine their hydrophilicity (Figure 2). The WCA decreased by 33% with the deposition of polydopamine only on the PVDF membrane, which was expected since it is widely reported that PDA increases the hydrophilicity of polymeric membranes [13,16,17]. An additional 34% decrease in the WCA occurred after the immobilization of the enzymes. Schmidt et al. [3] also reported a considerable decrease in the WCA after immobilizing a mixture of enzymes on a PVDF membrane (from 120 to 65°). The attached enzymes can increase the hydrophilicity of the membrane, possibly due to their hydrophilic functional groups such as amino acids with polar side chains (e.g., serine, threonine, asparagine, and glutamine). These groups interact with water molecules, increasing the overall hydrophilicity of the membrane surface [30].

**Figure 3:**
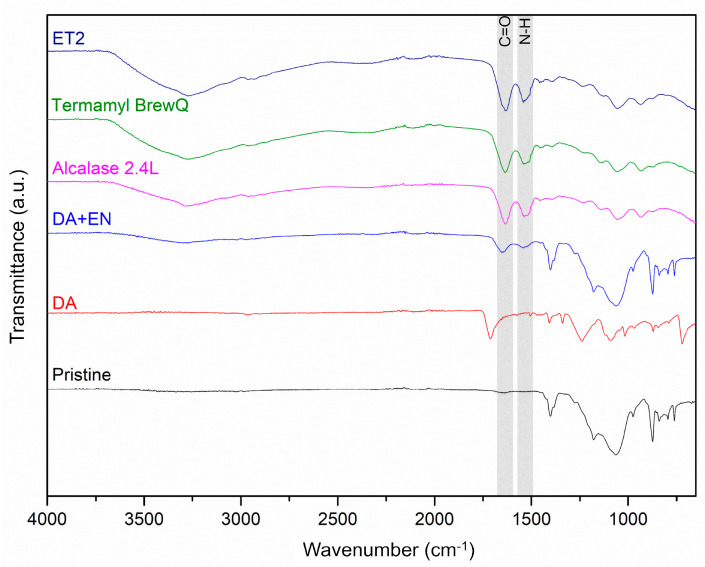
ATR-FTIR of the pristine and PDA-coated (DA, 0.2 mg/mL) membranes, the membrane with immobilized enzymes by PDA (DA + EN, 0.2 mg/mL of DA and 4 mg/mL of EN), and the lyophilized protease (Alcalase 2.4L), amylase (Termamyl BrewQ), and lipase (ET2).

Figure 3 shows the ATR-FTIR of the pristine and modified membranes with PDA only and PDA and the enzymes. The membrane with the immobilized enzymes displayed functional groups similar to those in the free enzymes including C=O stretching related to primary amides typically found in the proteins’ α-helix secondary structure (1650 cm^−1^) and N-H bending (1540 cm^−1^) of the secondary amides [7,31,32]. The PDA coating signals were not observed on the membrane with attached enzymes, likely due to the lower concentration of DA in relation to the concentration of enzymes during immobilization [7].

### 3.4. Filtration Experiments

**Figure 4:**
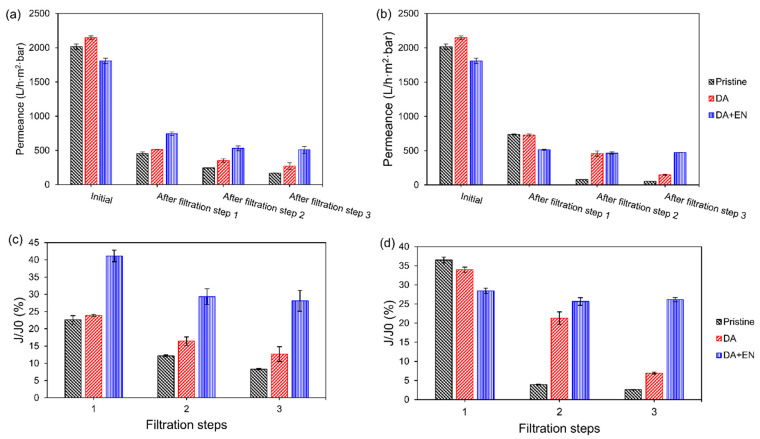
Initial pure water permeance of the pristine, PDA-coated (0.2 mg/mL), and PDA + EN (0.2 mg/mL of DA and 4 mg/mL of EN) modified membranes and pure water permeance after three consecutive filtration steps of (**a**) a mixture of starch (0.75 g/L), soybean oil (1 g/L), and casein (0.5 g/L), and (**b**) milk powder (2 g/L). Relative pure water flux of the membranes after each filtration step of the (**c**) mixture and (**d**) milk powder.

The membrane modified with 0.2 mg/mL of DA and 4 mg/mL of EN was compared to the membrane modified with only 0.2 mg/mL of DA and the pristine membrane. Figure 4 shows the pure water permeances of the membranes at the beginning of the process and at the end of each filtration step. The initial pure water permeance of the membrane increased after the PDA coating due to the increase in hydrophilicity (Figure 2). However, when the enzymes were immobilized on the membrane, the initial pure water permeance decreased despite the increase in hydrophilicity, which is an indication of pore size narrowing after modification. Proner et al. [33] also observed pore blockage after PDA and PEI co-polymerization on polyethersulfone ultrafiltration membranes. Although the initial pure water permeance was lower for the enzyme-modified membrane, its pure water permeance was higher after all filtration steps when the mixed solution was filtered (Figure 4a), which resulted in superior relative pure water fluxes (41.2 ± 1.7% for step 1, 29.3 ± 2.3% for step 2, and 28.1 ± 3.0% for step 3) compared to the PDA-coated membrane (23.9 ± 0.3%, 16.4 ± 1.2%, and 12.7 ± 2.1% for steps 1, 2, and 3, respectively) and the pristine membrane (22.6 ± 1.2%, 12.2 ± 0.3%, and 8.3 ± 0.2%, respectively). The higher fluxes could be due to the hydrolysis of the foulants during filtration and the higher hydrophilicity of the membrane with immobilized enzymes, as shown by the contact angle (Figure 2). Hydrophobic foulants, such as oils, greases, or hydrophobic organic compounds, tend to accumulate on the surface of hydrophobic membranes like PVDF. These interactions occur because both the membrane and the foulants minimize their exposure to water by adhering to each other. Therefore, a higher hydrophilicity reduces the hydrophobic interactions between membrane and foulants, making the membrane more prone to water permeation.

When a milk powder solution was filtered (Figure 4b), which characterizes a more complex feed representing simulated wastewater from the dairy industry, the differences between the membranes decreased. The enzymatically active membrane showed a lower pure water permeance after the first filtration step. However, it was able to keep the same permeance after all filtration cycles, which was not the case for the other membranes that showed significant decreases after each filtration. These results could also be observed for the relative pure water fluxes: the enzymatically active membrane showed a lower relative flux after the first filtration step (28.4 ± 0.7%) than the other membranes (36.5 ± 0.8% for the pristine membrane and 34.0 ± 0.7% for the PDA-coated membrane). However, it maintained its flux over the additional filtration steps (25.7 ± 1.0% after filtration step 2 and 26.1 ± 0.6% after filtration step 3), which was not observed for the membranes without the enzymes (the relative flux of the pristine membrane decreased to 3.9 ± 0.1% and 2.6 ± 0.1% after filtration steps 2 and 3, and the relative flux of the PDA-coated membrane decreased to 21.3 ± 1.6% and 6.9 ± 0.2%, respectively). These results suggest that the enzymes are active on the membrane surface, decreasing the fouling layer as it is deposited over the filtration. Therefore, the method used was successful in developing a fouling-degrading membrane with self-cleaning properties.

## 4. Conclusions

This research verified that the use of polydopamine to bond multiple enzymes on a polymeric membrane is possible. By optimizing the concentrations of dopamine hydrochloride (DA) and enzymes (EN) in the immobilization solution, the active membrane achieved hydrolytic activities up to 0.82 ± 0.03 mg_starch_/min·cm^2^ for amylase, 20.48 ± 0.37 µmol_p-nitrophenol_/min·cm^2^ for lipase, and 7.82 ± 0.33 nmol_tyrosine_/min·cm^2^ for protease when using 0.2 mg/mL of DA and 4 mg/mL of enzymes. The immobilization of the enzymes resulted in an increased water affinity compared to both the membrane modified with DA only and the pristine membrane. The enzymatically active membrane showed a high resistance to fouling (relative pure water flux limited to 26% after a simulated dairy wastewater filtration) due to the hydrolysis of the foulants and increased water affinity. Multienzyme immobilization using a PDA coating in only one step is a greener method than conventional techniques and can facilitate the scale-up of the process.

## Figures and Tables

**Table 1:** Protein content and hydrolytic activity of amylase, lipase, and protease in the crude extract and after dialysis and lyophilization.

Enzyme	Protein Content (mg/g)	Specific Hydrolytic Activity	Unit
Amylase—crude extract	41.65 ± 0.16	401.76 ± 15.80	g_starch_/min·g
Amylase—lyophilized	657.76 ± 0.83	402.34 ± 17.13	g_starch_/min·g
Lipase—crude extract	28.62 ± 2.65	6.43 ± 0.83	mmol_p-nitrophenol_/min·g
Lipase—lyophilized	536.02 ± 1.44	6.63 ± 0.24	mmol_p-nitrophenol_/min·g
Protease—crude extract	82.37 ± 1.23	9.29 ± 1.34	mmol_tyrosine_/min·g
Protease—lyophilized	599.10 ± 6.27	12.33 ± 1.02	mmol_tyrosine_/min·g

**Table 2:** CCRD 2^2^ results for the hydrolytic activities of amylase, lipase, and protease immobilized on the membrane by different concentrations of dopamine hydrochloride (DA) and enzymes (EN).

Assay	DA (mg/mL)	EN (mg/mL)	Amylase Activity (mg_starch_/min·cm^2^)	Lipase Activity (µmol_n-nitrophenol_/min·cm^2^)	Protease Activity (nmol_tyrosine_/min·cm^2^)
1	0.3	3.0	0.80	18.52	5.24
2	0.7	3.0	0.49	13.25	4.56
3	0.3	5.0	0.62	18.11	10.16
4	0.7	5.0	0.80	13.35	8.00
5	0.2	4.0	0.82	20.48	7.82
6	0.8	4.0	0.69	12.30	5.60
7	0.5	2.6	0.68	15.20	3.27
8	0.5	5.4	0.83	16.11	8.22
9	0.5	4.0	0.85	15.15	6.05
10	0.5	4.0	0.90	15.19	7.01
11	0.5	4.0	0.86	14.64	6.63

**Table 3:** Additional immobilization tests using the best conditions for each enzyme determined by the CCRD 2^2^. Note: According to Tukey’s test, different letters mean significantly different enzyme activities (*p* < 0.05).

DA (mg/mL)	EN (mg/mL)	Amylase Activity (mg_starch_/min·cm^2^)	Lipase Activity (µmol_p-nitrophenol_/min·cm^2^)	Protease Activity (nmol_tyrosine_/min·cm^2^)
0.3	5.0	0.62 ± 0.03 ^a^	18.11 ± 0.31 ^a^	10.16 ± 0.48 ^a^
0.2	4.0	0.82 ± 0.03 ^b^	20.48 ± 0.37 ^b^	7.82 ± 0.33 ^b^
0.5	4.0	0.87 ± 0.02 ^b^	14.99 ± 0.29 ^c^	6.56 ± 0.41 ^c^

## Data Availability

The original contributions presented in the study are included in the article and Supplementary Materials, further inquiries can be directed to the corresponding authors.

## Supplementary Materials

The following supporting information can be downloaded at: https://www.mdpi.com/article/10.3390/membranes14100208/s1, Table S1: Analysis of Variance (ANOVA) for the hydrolytic activity of amylase obtained in the CCRD 2²; Table S2: Analysis of Variance (ANOVA) for the hydrolytic activity of lipase obtained in the CCRD 2²; Table S3: Analysis of Variance (ANOVA) for the hydrolytic activity of protease obtained in the CCRD 2².
