# Supplementary material for: Multienzyme Immobilization on PVDF Membrane via One-Step Mussel-Inspired Method: Enhancing Fouling Resistance and Self-Cleaning Efficiency
Source: Membranes (Basel). 2024 Sep 27;14(10):208. doi: 10.3390/membranes14100208 (PMC11509426; doi:10.3390/membranes14100208)
Supplement: Supplementary file 1 [file membranes-14-00208-s001.zip › membranes-3209577-supplementary.pdf]

Supplementary Materials

Article

Multienzyme Immobilization on PVDF Membrane via One-Step Mussel-Inspired Method: Enhancing Fouling Resistance and Self-Cleaning Efficiency

Jéssica Mulinari [1,2,*], Diane Rigo [1,2], Carolina Elisa Demaman Oro [1,2], Alessandra Cristina de Meneses [1], Guilherme Zin [1], Rafael Vidal Eleutério [3], Marcus Vinícius Tres [4,*] and Rogério Marcos Dallago [2]

1 TransferTech Gestão de Inovação, Erechim 99700-420, Brazil; dianerigo@hotmail.com (D.R.); carolinae.oro@hotmail.com or 044079@aluno.uricer.edu.br (C.E.D.O.); acmeneses@transfertech.com.br (A.C.d.M.); gzin@transfertech.com.br (G.Z.)

2 Department of Food and Chemical Engineering, Universidade Regional Integrada do Alto Uruguai e das Missões (URI), 1621 Sete de Setembro Av., Centro, Erechim 99709-910, Brazil; dallago@uricer.edu.br

3 Graduate Program in Materials Science and Engineering (PGMAT), Federal University of Santa Catarina (UFSC), Florianópolis 88040-900, Brazil; rafaelvebr@gmail.com

4 Laboratory of Agroindustrial Processes Engineering (LAPE), Federal University of Santa Maria (UFSM), Cachoeira do Sul 96503-205, Brazil

* Correspondence: jessicamulinari15@gmail.com (J.M.); marcus.tres@ufsm.br (M.V.T.); Tel.: +55-54-996384542 (J.M.); +55-51-995582533 (M.V.T.)

Table S1. Analysis of Variance (ANOVA) for the hydrolytic activity of amylase obtained in the CCRD 2^2.

Variation Source	SS	DL	MS	$F_{calculated}$	F_{tabled}
Regression	0.15	5	0.031		
Residues	0.017	5	0.0033	9.37	5.05
Lack of fit	0.015	3	0.0051		
Pure error	0.0011	2	0.00053	9.66	19.16
Total	0.16	10			

Table S2. Analysis of Variance (ANOVA) for the hydrolytic activity of lipase obtained in the CCRD 2^2.

Variation Source	SS	DL	MS	$F_{calculated}$	F_{tabled}
Regression	61.54	5	12.31		
Residues	0.66	5	0.13	93.61	5.05
Lack of fit	0.47	3	0.16		
Pure error	0.19	2	0.094	1.68	19.16
Total	61.76	10			

Table S3. Analysis of Variance (ANOVA) for the hydrolytic activity of protease obtained in the CCRD 2^2.

Variation source	SS	DL	MS	$F_{calculated}$	F_{tabled}
Regression	35.12	5	7.02		
Residues	1.96	5	0.39	18.15	5.05
Lack of fit	1.47	3	0.49		
Pure error	0.47	2	0.23	2.10	19.16
Total	37.31	10			
